# Genetic Analysis and Prevalence Studies of the *brp* Exopolysaccharide Locus of *Vibrio vulnificus*

**DOI:** 10.1371/journal.pone.0100890

**Published:** 2014-07-11

**Authors:** Katherine L. Garrison-Schilling, Zelam M. Kaluskar, Bliss Lambert, Gregg S. Pettis

**Affiliations:** Department of Biological Sciences, Louisiana State University, Baton Rouge, Louisiana, United States of America; University of Helsinki, Finland

## Abstract

Phase variation in the Gram-negative human pathogen *Vibrio vulnificus* involves three colonial morphotypes- smooth opaque colonies due to production of capsular polysaccharide (CPS), smooth translucent colonies as the result of little or no CPS expression, and rugose colonies due to production of a separate extracellular polysaccharide (EPS), which greatly enhances biofilm formation. Previously, it was shown that the *brp* locus, which consists of nine genes arranged as an operon, is up-regulated in rugose strains in a c-di-GMP-dependent manner, and that plasmid insertions into the locus resulted in loss of rugosity and efficient biofilm production. Here, we have used non-polar mutagenesis to assess the involvement of individual *brp* genes in production of EPS and related phenotypes. Inactivation of genes predicted to be involved in various stages of EPS biosynthesis eliminated both the rugose colonial appearance and production of EPS, while knockout of a predicted flippase function involved in EPS transport resulted in a dry, lightly striated phenotype, which was associated with a reduction of *brp*-encoded EPS on the cell surface. All *brp* mutants retained the reduced motility characteristic of rugose strains. Lastly, we provide evidence that the *brp* locus is highly prevalent among strains of *V. vulnificus*.

## Introduction

*Vibrio vulnificus* is a Gram-negative bacterium found in estuarine and marine waters, and is commonly associated with human disease caused by ingestion of raw oysters or contact of the organism with an open wound. The mortality rate of *V. vulnificus* is the highest among food-borne pathogens, ranging from 50–75% [1], and pathogenesis is directly related to the presence of capsular polysaccharide (CPS), which protects the bacteria from the host immune system [2]–[5]. Encapsulated strains exhibit a smooth opaque colony phenotype on agar plates and kill an iron-overloaded mouse at lower doses than attenuated unencapsulated strains, which exhibit a smooth translucent phenotype [3]. A third colony type called rugose has been isolated from both opaque and translucent parental strains, and it is characterized by dry, wrinkled colonies, decreased motility, and robust biofilm formation caused by production of extracellular polysaccharide (EPS) [6], [7].

*V. vulnificus* can spontaneously switch among opaque, translucent and rugose phases in response to certain environmental conditions [8], [9]. Genetic loci relevant to these switching events include the group I CPS operon, involved in CPS biosynthesis and transport [10], [11], and the *brp* locus, which was shown to be involved in EPS production [7], [12]. The *brp* cluster (renamed from *wcr*) was originally identified by RT-PCR analysis as being more highly expressed in rugose variants when compared to opaque or translucent ones [7]. The translucent phenotype of a transposon mutant (TDB3[T]), which contains an insertion in the *brp* gene cluster, raised the possibility that one or more *brp* genes may also be required for CPS production [7], [13].

The *brp* locus is regulated by bacterial second messenger c-di-GMP, though the mechanism remains undetermined [12]. The importance of c-di-GMP as a regulator of EPS production, and biofilm formation has been established previously in several bacterial species [14], [15]. Recently, an additional exopolysaccharide locus, *rbd*, was characterized in *V. vulnificus*, and it was found to enhance biofilm and cell aggregation phenotypes, though its polysaccharide did not appear to be required for either development or maintenance of the rugose phenotype [16].

In this study, we used a non-polar mutagenesis approach to assess the involvement of individual *brp* genes in exopolysaccharide production and related phenotypes. Four *brp* genes were disrupted, and two phenotypes with respect to colony morphology and EPS production were observed. All non-polar *brp* mutants showed greatly reduced biofilm capability and also remained less motile than opaque or translucent variants. Through a combined PCR and Southern blotting approach, we also found the *brp* locus to be widespread within this species.

## Materials and Methods

### Bacterial strains & growth conditions

All *V. vulnificus* strains were grown in heart infusion broth (Difco) supplemented to 2% NaCl (HI) and on HI agar plates containing 18 g/l of agar (Difco). Broth cultures were incubated at 30°C and 200 rpm; plates were incubated overnight (ON) for 16–24 h at 30°C. Phase switching assays in HI and growth curves were all performed as previously described [8]. *Escherichia coli* strains were grown in LB broth (Difco), broth cultures were incubated at 37°C and 250 rpm, and plates were incubated ON for 16–24 h at 37°C. Antibiotics (Sigma) were used at the following concentrations: 150 µg/ml kanamycin, 50 µg/ml ampicillin, and 2 µg/ml chloramphenicol for *V. vulnificus* and 50 µg/ml kanamycin, 50 µg/ml ampicillin, and 10 µg/ml chloramphenicol for *E. coli*. Arabinose (Sigma) was typically added to a final concentration of 0.2% when needed. *E. coli* and *V. vulnificus* strains used or created in this study are listed in Table 1.

**Table 1:** Strains used in this study.

	Strain	Genotype/Description	Source or Reference
*E. coli*			
	BRL2288	F^−^ *araD139* Δ*(ara-leu)7679* Δ*(lac)X74 galU galK hsdR2* (r_κ_^−^ m_κ_^−^) *mcrB1 rpsL recA56* (Str^r^)	[36]
	S17.1	*thi pro hsdR hdsM^+^ recA* RP4-2-Tc::Mu-Km-Tn7	[37]
	SY327λpir	Δ(*lac pro*) *argE*(Am) *rif malA recA56* λ*pir*	[38]
	S17.1λpir	*thi pro hsdR hdsM^+^ recA* RP4-2-Tc::Mu-Km-Tn7 λ*pir*	[37]
*V. vulnificus*			
	1003(O)	formerly 1003; wound isolate from Louisiana, opaque	[39]
	BG(R)	spontaneous rugose isolate of 1003(O)	[6]
	AZ(T)	spontaneous translucent isolate of 1003(O)	[32]
	ABZ1(T)	transposon mutant of 1003(O) (*wcvA*::mini-Tn*10)*, TrS	[32]
	ABZ1(R)	spontaneous rugose isolate of ABZ1(T)	[6]
	TDB3(T)	transposon mutant of 1003(O) (*brpI*::mini-Tn*10)*, TrS	[13]
	YJ016	clinical isolate from Taiwan, opaque	[40]
	KG3(R)	spontaneous rugose isolate of YJ016	This study
	KG4(T)	spontaneous translucent isolate of YJ016	This study
	KG3-17	non-polar *brpI* mutant of KG3(R), Km^R^	This study
	KG3-01	non-polar *brpA* mutant of KG3(R), Km^R^	This study
	KG3-02	non-polar *brpD* mutant of KG3(R), Km^R^	This study
	KG3-03	non-polar *brpJ* mutant of KG3(R), Km^R^	This study
	KG3-17C	KG3-17 complemented with *brpI* gene on pVV40, Km^R^, Chl^R^	This study
	KG3-01C	KG3-01, complemented with *brpA* gene on pVV43, Km^R^, Chl^R^	This study
	KG3-02C	KG3-02, complemented with *brpD* gene on pVV41, Km^R^, Chl^R^	This study
	KG3-03C	KG3-03, complemented with *brpJ* gene on pVV45, Km^R^, Chl^R^	This study
	TDB3(T)C1	TDB3(T), complemented with *brpI* gene on pVV40, Km^R^, Chl^R^	This study
	TDB3(T)C2	TDB3(T), complemented with *brpIJK* genes on pVV53, Km^R^, Chl^R^	This study
	YJ-10	non-polar *brpJ* mutant of YJ016, Km^R^	This study
	1001	blood isolate, opaque	[39]
	1004	stool isolate, opaque	[39]
	1005	blood isolate, opaque	[39]
	1007	blood isolate, opaque	[39]
	1009	blood isolate, opaque	[39]
	1014	blood isolate, opaque	[39]
	1456(O)	unknown origin, opaque	Lab collection
	1456(T)	spontaneous translucent variant of 1456(O)	Lab collection
	1657(O)	unknown origin, opaque	Lab collection
	1657(T)	spontaneous translucent variant of 1657(O)	Lab collection
	95-10-15	unknown origin, opaque	Lab collection
	96-7-155	eel isolate, opaque	Lab collection
	ATCC27562	clinical isolate, opaque	[41]
	C7184	clinical isolate, opaque	[42]
	CMCP6	clinical isolate, opaque	[43]
	CP-Clam-2	oyster isolate, opaque	Lab collection
	CP-Mussel-10	oyster isolate, opaque	Lab collection
	CP-Sed-5	sediment isolate, opaque	[8]
	F8Oyster11(O)	oyster isolate, opaque	Lab collection
	F8Oyster11(T)	spontaneous translucent variant of F8Oyster11(O)	Lab collection
	MLT124	oyster isolate, opaque	[8]
	MLT136(O)	formerly MLT136; oyster isolate, opaque	[8]
	MLT136(T)	spontaneous translucent isolate of MLT136(O)	Lab collection
	MLT141	oyster isolate, opaque	Lab collection
	MLT198	sediment isolate, translucent	Lab collection
	132a1	environmental isolate, opaque	M.E. Janes
	132z2	environmental isolate, opaque	M.E. Janes
	212b6	environmental isolate, opaque	M.E. Janes
	212f12	environmental isolate, opaque	M.E. Janes
	212s7	environmental isolate, opaque	M.E. Janes
	342e9	environmental isolate, opaque	M.E. Janes
	342s6	environmental isolate, opaque	M.E. Janes
	NOLA18	environmental isolate, New Orleans, LA, opaque	Lab collection
	212f18	environmental isolate, opaque	M.E. Janes

### Molecular genetic and recombinant DNA techniques

DNA manipulations were carried out using standard molecular techniques [17]. Restriction enzymes, calf intestinal alkaline phosphatase (CIP), T4 polynucleotide kinase, and Klenow polymerase were obtained from New England Biolabs, Pfu polymerase from Stratagene, AmpliTaq polymerase from Applied Biosystems, and primers from Sigma Genosys. Plasmids used or created in this study are listed in Table 2, while primers are listed in Tables 3 and 4. Genomic DNA was isolated and PCRs for *brp* gene linkage analysis were completed as described [7], [8]. For Southern blotting, fragments specific for the *brpC* or *brpI* genes were generated via PCR with primer pairs RUG17/RUG18, and CAP27/CAP28, respectively. Production of radiolabeled probes and hybridizations were performed as described [7] using ca. 10^8^ cpm/ml of probe per hybridization.

**Table 2:** Plasmids used in this study.

Plasmid	Description	Reference/source
**Vectors**		
pSP72	cloning vector, Ap^R^	Promega
pGP704sacB28	suicide vector, Ap^R^	[19]
pBBR1MCS	broad host range cloning vector, Chl^R^	[21]
pACW29	contains the 840bp nonpolar Km^R^ cassette inserted in *wza*_Vv_, Km^R^	[11]
pKan2	contains the 840bp nonpolar Km^R^ cassette of pACW29 cloned into pSP72, Km^R^	This study
pBAD/His A	expression vector containing the *araC* gene and P_BAD_ promoter, Ap^R^	Invitrogen
pBBRBAD1	P_BAD_ promoter and *araC* gene cloned into pBBR1MCS, Chl^R^	This study
pBBRBAD2	P_BAD_ promoter and *araC* gene in the opposite orientation of pBBRBAD1, Chl^R^	This study
**Non-polar mutagenesis clones**		
pVV4	*brpI* fragment cloned into pSP72, Ap^R^	This study
pVV8	nonpolar Km^R^ cassette inserted in the *brpI* fragment of pVV4, Ap^R^, Km^R^	This study
pVV18	*brpI*-Km^R^ cassette fusion from pVV8 cloned into pGP704sacB28, Ap^R^, Km^R^	This study
pVV1	*brpD* fragment cloned into pSP72, Ap^R^	This study
pVV2	nonpolar Km^R^ cassette inserted in the *brpD* fragment of pVV1, Ap^R^, Km^R^	This study
pVV21	*brpD*-Km^R^ cassette fusion from pVV2 cloned into pGP704sacB28, Ap^R^, Km^R^	This study
pVV27	*brpA* deletion (*ΔbrpA*) fragment cloned into pSP72, Ap^R^	This study
pVV28	nonpolar Km^R^ cassette inserted in the *ΔbrpA* fragment of pVV27, Ap^R^, Km^R^	This study
pVV29	*ΔbrpA*-Km^R^ cassette fusion from pVV28 cloned into pGP704sacB28, Ap^R^, Km^R^	This study
pVV42	*brpJ* deletion (*ΔbrpJ*) fragment cloned into pSP72, Ap^R^	This study
pVV44	nonpolar Km^R^ cassette inserted in the *ΔbrpJ* fragment of pVV42, Ap^R^, Km^R^	This study
pVV46	*ΔbrpJ*-Km^R^ cassette fusion from pVV44 cloned into pGP704sacB28, Ap^R^, Km^R^	This study
**Complementation/overexpression clones**		
pVV40	*brpI* gene cloned into pBBRBAD2, Chl^R^	This study
pVV41	*brpD* gene cloned into pBBRBAD2, Chl^R^	This study
pVV43	*brpA* gene cloned into pBBRBAD1, Chl^R^	This study
pVV45	*brpJ* gene cloned into pBBRBAD2, Chl^R^	This study
pVV53	*brpIJK* genes cloned into pBBRBAD2, Chl^R^	This study

Ap^R^, ampicillin resistant; Km^R^, kanamycin resistant, Chl^R^, chloramphenicol resistant.

**Table 3:** Primers used for non-polar mutagenesis & complementation experiments.

Primer	Sequence (5′→3′)^1^
Npm01	AAAAAGAATTCCGACGAGGCCTTCTGTGGTG
Npm02	AAAAAGATATCCGAAGTGAGGCGTCCCGGTAG
Npm03	AAAAACTCGAGGTAAGAGGCATACCTTCC
Npm04	AAAAAGAATTCTACGATCTGCCACTCTAC
Del_brpA_A	AAAGAATTCACGCCACTTTGGTAGGAG
Del_brpA_B	*GTCCATTTA* ***CCCGGG*** *ACAAGC*CCGTGTTTAAAGGCTTTACTGG
Del_brpA_C	*GCTTGT* ***CCCGGG*** *TAAATGGAC*TCATGTGAGCGAATCAATCC
Del_brpA_D	AAAGGATCCCAGATGCGTGCCCAGATTAC
Del_brpJ_A	AAAGAATTCGCGCCAATTTCAGGCAAGGTAG
Del_brpJ_B	*CTTCAAT* ***CCCGGG*** *GATCCAG*CTTAGGCTTTGTACCCATCG
Del_brpJ_C	*CTGGATC* ***CCCGGG*** *ATTGAAG*CTCATGGGATAACCCAGAC
Del_brpJ_D	AAATCTAGAAAGCATTTGGCGGTGGTGTAC
brpA-For	AAACTCGAGCCCACCCACACGGGTAACAAAC
brpA-Rev	AAAAAGCTTAACGACACTCAAGGGCGGTAGC
Npm11	AAAAATCTAGATGATTAAGCACGCTGAGC
Npm12	AAAAACTGCAGCAGAAAAGGAGAGAGCTC
Npm13^2^	AAAAAGGATCCCTTCGCCAGTAACGAGGTCG
brpJ-For	AAACCCGGGAAGATTGGCGCTCAGCAGCC
brpJ-Rev	AAATCTAGAACTGTCATGACGCAAATGAC
brpK-For	AAAAATCTAGAGCAATGGCGATGTCTTGCTG
brpIJK-Rev	AAAAACCCGGGCTAAAGACACCAGGGACGAG

1 Restriction enzyme sites are underlined. Homologous sequence used in SOE is italicized. *Sma*I sites are in bold.

2 From Grau, et al. 2008 [7].

**Table 4:** Primers used for *brp* distribution analysis.

Primer	Sequence (5′→3′)	Description
RUG13^1^	CGAGAGAGATGAGAGCAATG	Used to demonstrate *brpA-brpB* linkage
RUG16^1^	TTCCTCTCGCAATACGCCAC	Used to demonstrate *brpA-brpB* linkage
RUG15^1^	CAAGAGCATACGACCTGGAC	Used to demonstrate *brpB-brpC* linkage
RUG18^1^	TGGCGAGGATGACTTATCAA	Used to demonstrate *brpB-brpC* linkage
RUG19^1^	ACGCAGACCTCCACTAGAGA	Used to demonstrate *brpD-brpF* linkage
RUG34^1^	GAAGGTGCAGGAAGAGTTGA	Used to demonstrate *brpD-brpF* linkage
CAP25^1^	GTAGAGTGGCAGATCGTACA	Used to demonstrate *brpH-brpI* linkage
CAP28^1^	TGTCACGCAACTGCTGTTCT	Used to demonstrate *brpH-brpI* linkage
CAP27^1^	TACCAATCTAACGCCGGCAA	Used to demonstrate *brpI-brpJ* linkage (also used with CAP28 to generate *brpI* probe)
RUG45^1^	AATCCAATGAGCAGCGAAGTCC	Used to demonstrate *brpI-brpJ* linkage
RUG43	AGCCAGAAGATAGCCTGTC	Used to demonstrate *brpJ-brpK* linkage
RUG46^1^	AGCGATACGCCATGTTACTCC	Used to demonstrate *brpJ-brpK* linkage
RUG33^1^	GAAGGTGCAGGAAGAGTTGA	Used to generate *brpF* probe (with RUG34)
RUG44	GGAGTCGAGCTCTTCATTCC	Used to generate *brpK* probe (with RUG43)
RUG17	CGGAACTTATTAATCGAGAC	Used with RUG18 to generate *brpC* probe
wza-For^2^	GGTGCTCTACGCGTTAAAACG	Used to amplify *wzb* gene
wzc-Rev^2^	GAGTTATCAACGGAAGATCGG	Used to amplify *wzb* gene

1 From Grau, et al. 2008 [7].

2 From Garrison-Schilling, et al. 2011 [8].

### Generation of in-frame *brpD* and *brpI* insertion mutants

Mutants of *brpD* and *brpI* were generated from the rugose parental strain KG3(R) as follows. Using PCR, ∼1-kb fragments of *brpD* and *brpI* were amplified using primer pairs Npm1/Npm2 and Npm3/Npm4, respectively. Each 50-µl PCR reaction mixture contained 5 µl of 10× buffer, 4 µl of a 10 mM dNTP mixture (each dNTP at 2.5 mM), 1 µl of each primer (20 µM), 1 µl of Pfu polymerase (2.5 U/µl), 100 ng of YJ016 genomic DNA, and nuclease-free H_2_O. The PCRs were performed using an initial temperature of 95°C for 2 min, followed by 30 cycles of 95°C for 45 sec, 55°C for 45 sec, and 72°C for 1.5 min; these cycles were then followed by a final extension at 72°C for 10 min and holding at 4°C. PCR mixtures were examined by gel electrophoresis, and, upon relevant restriction enzyme digestion, the 1-kb products were cloned into the *Eco*RI and *Eco*RV sites and the *Eco*RI and *Xho*I sites of vector pSP72 (Promega) to create pVV1 (*brpD*) and pVV4 (*brpI*), respectively. An 840-bp nonpolar kanamycin-resistance cassette [18], which was generated by digestion of plasmid pKan2 with *Sma*I, was cloned in the correct orientation into the *Cla*I site (which had been blunt ended using Klenow) of pVV1 or the *Msc*I site of pVV4 to create pVV2 and pVV8, respectively. To confirm the cassette was inserted in frame with the downstream portion of *brpD* and *brpI*, sequencing reactions were performed using BigDye v3.1 (Applied Biosystems) [8]. The 1.88-kb *Hpa*I-*Sma*I fragment of pVV2 and the 1.75-kb *Eco*RI-*Xho*I fragment (whose ends were filled in using Klenow) of pVV8 were then cloned into the blunt-ended *Xba*I site of suicide vector pGP704*sacB*28 [19] to create pVV21 (*brpD*) and pVV18 (*brpI*). Matings between *V. vulnificus* KG3(R) and S17.1λ*pir* harboring the respective insertion plasmids were carried out on filter membranes; kanamycin-resistant, ampicillin-sensitive transconjugants that had undergone double homologous recombination were selected, and PCRs using AmpliTaq polymerase and primer pairs Npm1/Npm2 (*brpD*) and Npm3/Npm4 (*brpI*) were performed as described [7] to check for proper integration of the cassette. Southern blot hybridizations were used to confirm the mutant strains KG3-02 (*brpD::aphA-3*) and KG3-17 (*brpI::aphA-3*).

### Generation of in-frame *brpA* and *brpJ* deletion mutants

Deletions of *brpA* and *brpJ* were generated from the rugose strain KG3(R) as follows. Using Pfu polymerase and PCR conditions as described above, primers A and B amplified a 1-kb fragment at the 3′ end of the target gene (AB), while primers C and D amplified a 1-kb fragment at the 5′ end (CD). The resulting 1-kb AB & CD fragments for a given gene were joined using splice overlap extension with primers A and D as previously described [20] to create a 2-kb deletion fragment. The fragment specific for *brpA* was digested with *Eco*RI and *Bam*HI and cloned into these sites on pSP72, creating pVV27, while the *brpJ*-specific fragment was digested with *Eco*RI and *Xba*I and cloned into these sites on pSP72 to create pVV42. The 840-bp nonpolar kanamycin cassette was then cloned as a *Sma*I fragment in the correct orientation into the *Sma*I site of each deletion clone to create pVV28 (*brpA*) and pVV44 (*brpJ*). Following sequencing as described earlier, the *brpA* deletion construct containing the non-polar cassette was cloned as a 3-kb *Eco*RI-*Hind*III fragment (made blunt ended with Klenow) into the blunt-ended *Xba*I site of pGP704*sacB*28 to create pVV29, while the same construct for *brpJ* was cloned as a 3-kb *Cla*I-*Xho*I fragment (with ends filled) into the blunt-ended *Xba*I site of pGP704*sacB*28 to create pVV46. Intergeneric matings, selection of transconjugants and subsequent PCR analysis of potential mutants were performed as described in the previous section, except that primer pairs Del_wcrA_A/Del_wcrA_D (*brpA*) and Del_wcrJ_A/Del_wcrJ_D (*brpJ*) were used for the latter step. Southern blot hybridizations were used to confirm the mutant strains KG3-01 (*ΔbrpA::aphA-3*) and KG3-03 (*ΔbrpJ::aphA-3*).

### Complementation of *brp* mutants

Complementation vectors pBBRBAD1 and pBBRBAD2 were created by cloning the *Nco*I-*Sph*I fragment of pBAD1/His (Invitrogen), which contains the *araC* gene and arabinose-inducible promoter P_BAD_, in either orientation into the broad-host-range vector pBBR1MCS [21]. The *brpA, brpD, brpI, brpJ*, and *brpIJK* genes were each amplified from YJ016 via PCR with primer pairs wrcA-for/wcrA-Rev, Npm2/Npm13, Npm11/Npm12, wcrJ-For/wcrJ-Rev, and wcrK-For/wcrIJK-Rev, respectively. Following relevant restriction enzyme digestion, the amplified products were cloned into the *Xho*I and *Hind*III, *Eco*RV and *Bam*HI, *Xba*I and *Pst*I, *Sma*I and *Xba*I, and *Sma*I and *Xba*I sites of pBBRBAD1 or pBBRBAD2 to create the respective plasmids pVV43 (*brpA*), pVV41 (*brpD*), pVV40(*brpI*), pVV45(*brpJ*), and pVV53 (*brpIJK*). Matings between the mutant strains and S17.1 harboring the respective complementation plasmids were carried out as described earlier, and transconjugants were selected with chloramphenicol. Complementation was typically assessed by adding 0.2% arabinose to the growth media.

### Microscopy

Whole colony images were taken using a Leica MZ7.5 stereomicroscope and a SPOT Insight camera (Diagnostic Instruments, Inc.). Scanning electron microscopy (SEM) analysis of colony surfaces was performed as previously described [7].

### EPS isolation and visualization

EPS was isolated as previously described [22] with modifications. Two related protocols were performed with similar results. In the first protocol, a single colony was used to inoculate an entire plate of HI (with necessary antibiotics), which was incubated at room temperature (RT) for 2 days (2 plates were used for strain KG3-03 due to its relatively slow growth). Each lawn was collected by scraping with a glass pipette in the presence of PBS, and the resulting suspension was stirred briskly for 1-2 h at RT and then vigorously vortexed for 30 sec. Cells were harvested by centrifugation at 8,000 rpm for 1 h at 4°C, and a 5-ml aliquot of the supernatant was treated with 50 µg/ml RNase A (Qiagen) and 50 µg/ml DNase (Promega) in the presence of 10 mM MgCl_2_ at 37°C for 8 h, followed by treatment with 200 µg/ml protease (Sigma) at 37°C for 17.5 h. The supernatant was then extracted twice with an equal volume of phenol:chloroform (50∶50), and EPS was precipitated by the addition of 2 volumes of 100% ethanol. EPS was pelleted in a microfuge at 10,000 rpm for 30 min at 4°C, and the pellet was washed with 70% ethanol, dried and finally resuspended in 100 µl dH_2_O. An equal volume of each sample was then analyzed by SDS-polyacrylamide gel electrophoresis (PAGE) (100 V, 2–3 h) using 4% stacking/10% resolving gels. Finished gels were stained with Stains-All as previously described [23]. In the second protocol, a single colony was used to inoculate plates as before, except that 3 plates were used per strain. Following incubation, the lawns for a given strain were collected by scraping, the resulting suspension was vortexed vigorously for 1 min, and a 20-µl aliquot was used to create serial dilutions, which were plated on HI (with necessary antibiotics) to calculate CFU/ml of the cell suspension. The suspension was then shaken at 30°C for 90 min, and the vortexing and shaking was repeated a second time. Cells were harvested by centrifugation at 5,000 rpm for 15 min at 25°C, and the supernatant was re-centrifuged using the same parameters. The resulting supernatant was treated with RNaseA, DNase and protease, extracted twice with phenol:chloroform (50∶50) and polysaccharide was precipitated with 100% ethanol, all as in the first protocol. SDS-PAGE and staining were performed also as before, except that sample volumes were adjusted for differences in CFU/ml of the original cell suspensions.

### Pellicle and biofilm assays

Pellicle formation was observed after ON growth at 30°C in 3 ml HI (with necessary antibiotics and/or arabinose) with shaking at 200 rpm. Pellicle thickness was scored qualitatively as — (no pellicle), + (thin pellicle), ++ (pellicle), or +++ (thick pellicle). Three pellicles for each strain were scored. Biofilm assays were conducted as previously described [8], except static cultures were incubated for 18 h. At least two independent assays with six replicates of each strain were completed.

### Motility assays

Motility assays were conducted as previously described [6]. At least two independent assays with four replicates of each strain were completed.

### Statistical Analysis

All data were analyzed using a non-paired Student's t-test.

## Results

### Phenotypic characterization of individual *brp* mutants

**Figure 1:**
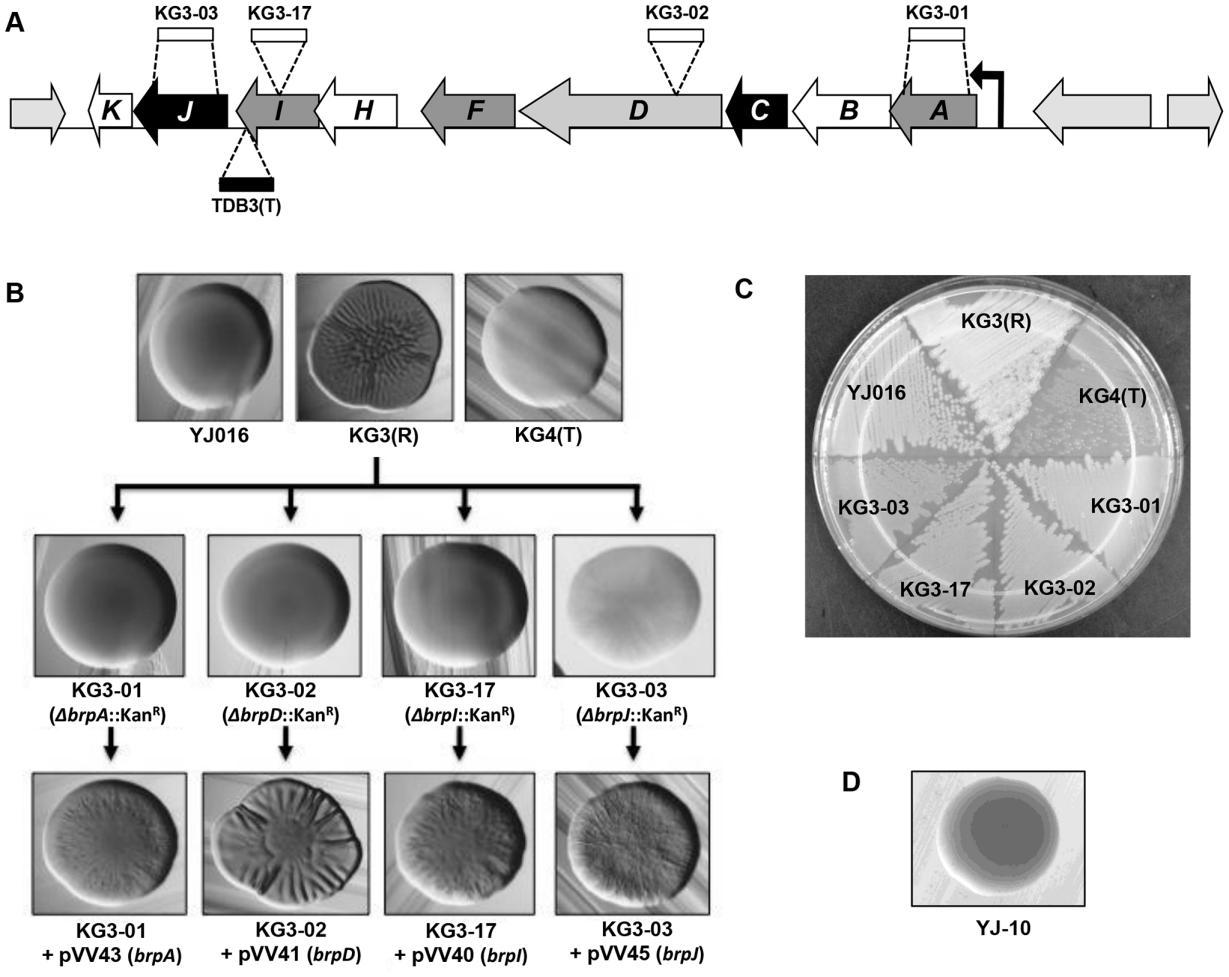
Phenotypes of individual *brp* gene mutants. **A**. Diagram of mutant construction for 4 *brp* genes. Open rectangles indicate the non-polar Kan^R^ cassette, while the filled rectangle indicates the mini-Tn*10* insertion in strain TDB3(T) (41). Shading of arrows for the *brp* cluster indicates putative function encoded: black, flippase involved in EPS transport (for *brpJ*) or EPS export-related protein (for *brpC*); light grey, tyrosine autokinase involved in EPS biosynthesis; dark grey, glycosyltransferase involved in EPS biosynthesis; white, unknown function. **B**. Colony morphology of opaque, rugose, and translucent control variants, the 4 *brp* mutant strains derived from strain KG3(R), and the complemented mutants. Strains were streaked for isolation on HI agar (containing kanamycin, chloramphenicol and arabinose, as appropriate) and incubated at 30°C ON. **C**. Streak plate of opaque, rugose, and translucent control variants and the 4 *brp* mutant strains. Strains were inoculated into HI broth (with kanamycin, where appropriate), shaken ON at 30°C, streaked onto HI (with no antibiotics), and incubated ON at 30°C. **D**. Colony morphology of the YJ016-derived *brpJ* mutant YJ-10. The strain was streaked for isolation on HI agar containing kanamycin and incubated at 30°C ON.

Given the possibility of co-transcription of the *brp* gene cluster (Figure 1A), a mutagenesis approach involving insertion of a non-polar kanamycin resistance cassette [18] was used in a rugose background to assess the contribution of select *brp* genes to rugosity, biofilm formation, exopolysaccharide production and motility. As shown previously, the presence of conserved domains revealed putative function for some of the *brp* genes while, for others, no conserved domains were apparent [7]; here, we chose to mutate four genes for which putative functions had been identified (Figure 1A). For the first two mutants, the kanamycin-resistant cassette was inserted between encoded functional domains of the *brpD* and *brpI* genes. Though, as detailed below, this approach yielded clear mutant phenotypes consistent with previous plasmid insertions into the locus [12], we were concerned that continued use of such a strategy would leave open the possibility of creating a truncated product which retained activity; thus, for the *brpA* and *brpJ* mutants, a different approach was employed which resulted in nearly the entire gene being replaced by the cassette (Figure 1A).

The *brpA* [KG3-01], *brpD* [KG3-02], and *brpI* [KG3-17] mutants lost the dry, wrinkled colony morphology of the KG3(R) parent, but appeared opaque, indicating they retained the ability to make CPS (Figure 1B–C). Meanwhile, the *brpJ* mutant (KG3-03) also lost its wrinkled look, though it remained dry in appearance with faint striations that became more pronounced upon extended incubation; thus, it possessed an intermediate phenotype which did not match any of the typical opaque, translucent or rugose colony types (Figure 1B–C). KG3-03 also grew detectably slower (by ∼50%) relative to the parent and other mutants (data not shown). It is unknown to what extent, if any, this defect contributed to the unique colonial phenotype of this mutant. Each mutant was then complemented using a cloned arabinose-inducible promoter construct which could induce expression of a wild type copy of each gene. The rugose colony phenotype was restored for each complemented mutant, although the degree of rugosity was clearly greater for KG3-02 containing the *brpD* complementation vector pVV41 (Figure 1B).

The complementation shown in Figure 1B was performed on plates containing 0.2% arabinose. When the concentration of arabinose was titrated (i.e., 0.02% and 0.002%), the strongest complementation was still always seen for KG3-02 (pVV41) (data not shown). Despite this effect, when pVV41 was introduced into strain KG3(R), rugosity of the parental strain did not appear to be increased relative to KG3(R) itself or KG3(R) containing the *brpI* complementation vector pVV40 (data not shown).

The opaque phenotype of the *brpI* mutant KG3-17 was consistent with a previous plasmid insertion into that gene [12] but not with the translucent phenotype of mutant TDB3(T) [13]. This mutant was derived from an opaque strain and contains a mini-Tn*10* insertion in the *brpI* gene. Upon further analysis of TDB3(T), we found here that it could not be complemented back to opacity with either the *brpI* gene alone (plasmid pVV40), or with *brpI* and the downstream *brpJ* and *brpK* genes (plasmid pVV53) all together (data not shown). Thus, it was unlikely that *brp* gene disruption was the cause of the translucent phenotype of TDB3(T). To further explore the genetic basis of this mutant, PCR was used to assess its *wzb* gene. It has been shown that deletions in the *wzabc* region of the group I CPS operon can cause an irreversible switch from the opaque to the translucent phenotype [10]. The *wzb* gene was still present with no mutations (data not shown), suggesting the translucent phenotype of TDB3(T) is the result of a separate genetic or epigenetic change somewhere in the genome.

**Figure 2:**
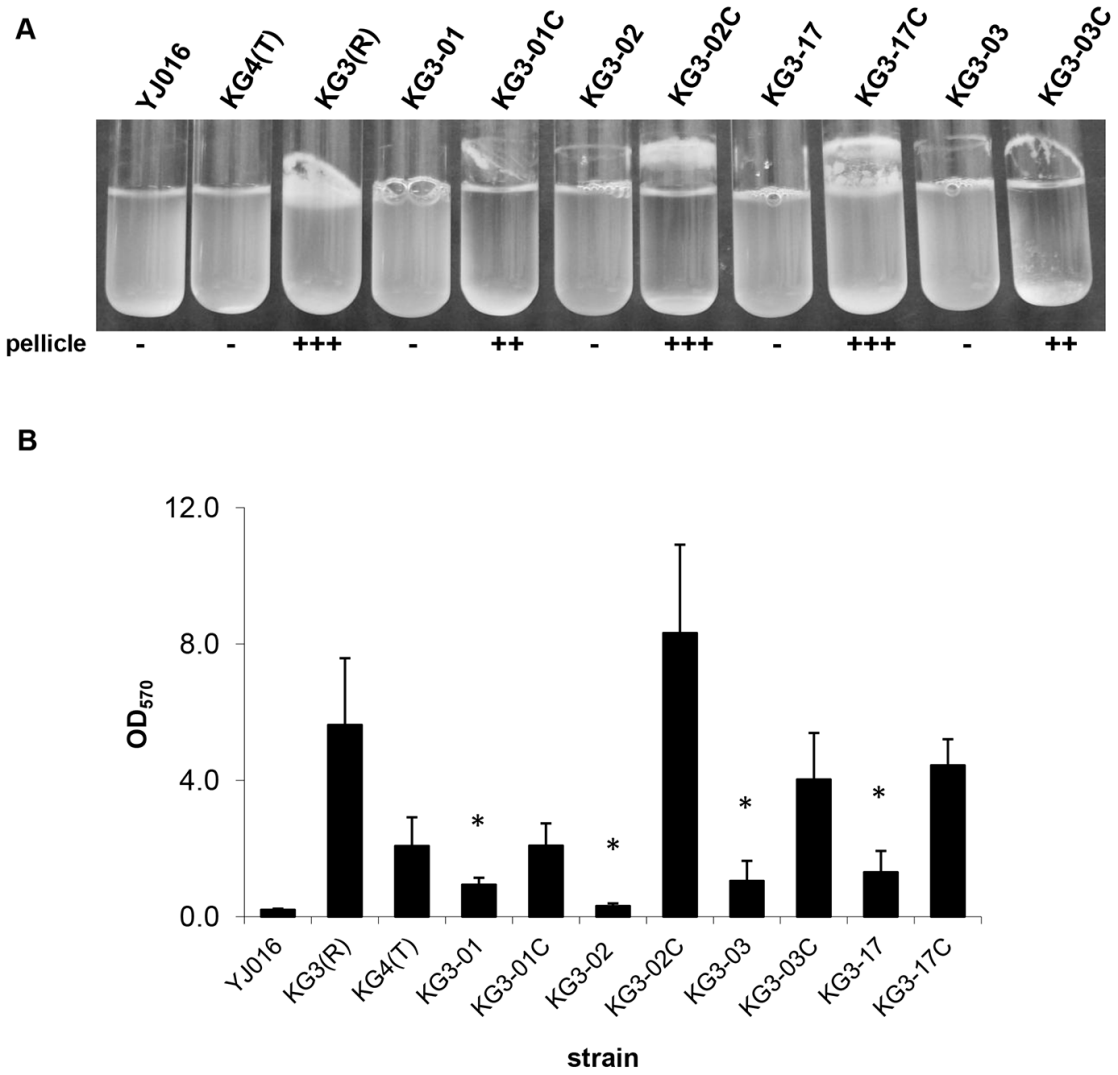
Biofilm formation by individual *brp* gene mutants. Biofilm formation was assessed qualitatively and quantitatively for opaque, rugose, and translucent control variants, the 4 *brp* mutant strains, and the complemented mutants. **A**. Following ON growth with shaking, 3 cultures per strain were assessed for pellicle formation and a representative is pictured. Pellicle thickness was scored qualitatively as — (no pellicle), + (thin pellicle), ++ (pellicle), or +++ (thick pellicle). **B**. Biofilm assays were performed on at least 6 independent culture replicates of each statically grown strain and OD_570_ values, which correspond to the amount of crystal violet staining of biofilm material, were averaged. Averages ± standard deviations (SD) are pictured here. Asterisk denotes *p*<0.001 versus KG3(R).

Since rugose isolates form robust biofilms [6], we expected that the *brp* mutants isolated here would produce less biofilm. Biofilm formation was assessed both qualitatively (pellicle formation) and quantitatively (biofilm assays). All four *brp* mutants lost the ability to form a pellicle, and pellicle formation was partially or fully restored upon complementation (Figure 2A). In quantitative biofilm assays, all four mutants formed significantly less biofilm than their rugose parent KG3(R) (max *p*<0.001), and biofilm formation was again partially or fully restored upon complementation (Figure 2B).

**Figure 3:**
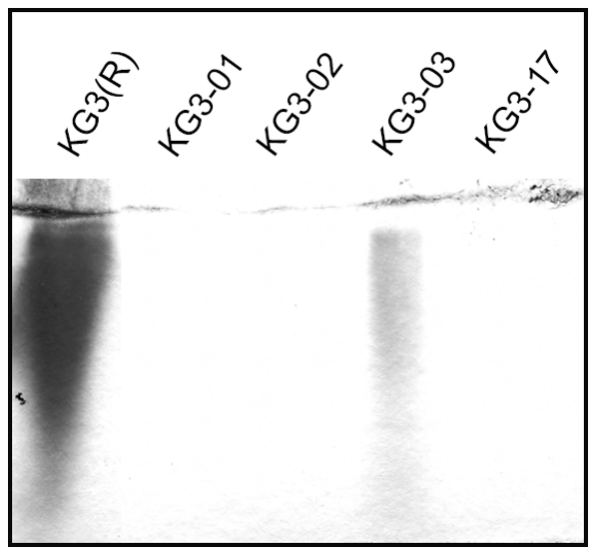
Evidence of EPS production for *brpJ* mutant strain KG3-03. An EPS extraction procedure (see Materials and Methods for details) was performed on KG3(R) and the 4 *brp* mutant strains, and the results were analyzed by SDS-PAGE using 4% stacking/10% resolving gels and subsequent staining with Stains-All.

We hypothesized that the intermediate phenotype of the *brpJ* mutant KG3-03 may be associated with a partial reduction, rather than complete loss, of *brp*-encoded EPS on the cell surface. Consistent with this view, a non-polar *brpJ* mutant of the opaque strain YJ016 (YJ-10) remained opaque (Figure 1D), indicating that deletion of *brpJ* per se does not appear to affect CPS production. To assess relative EPS production of KG3-03 directly, we compared the amount of EPS isolated from rugose strain KG3(R) and its four *brp* mutant derivatives. The data in Figure 3 show that much less EPS was recovered from KG3-03 cells than KG3(R), but reproducibly more than the other *brp* mutants, which did not yield detectable amounts of EPS in this analysis.

**Figure 4:**
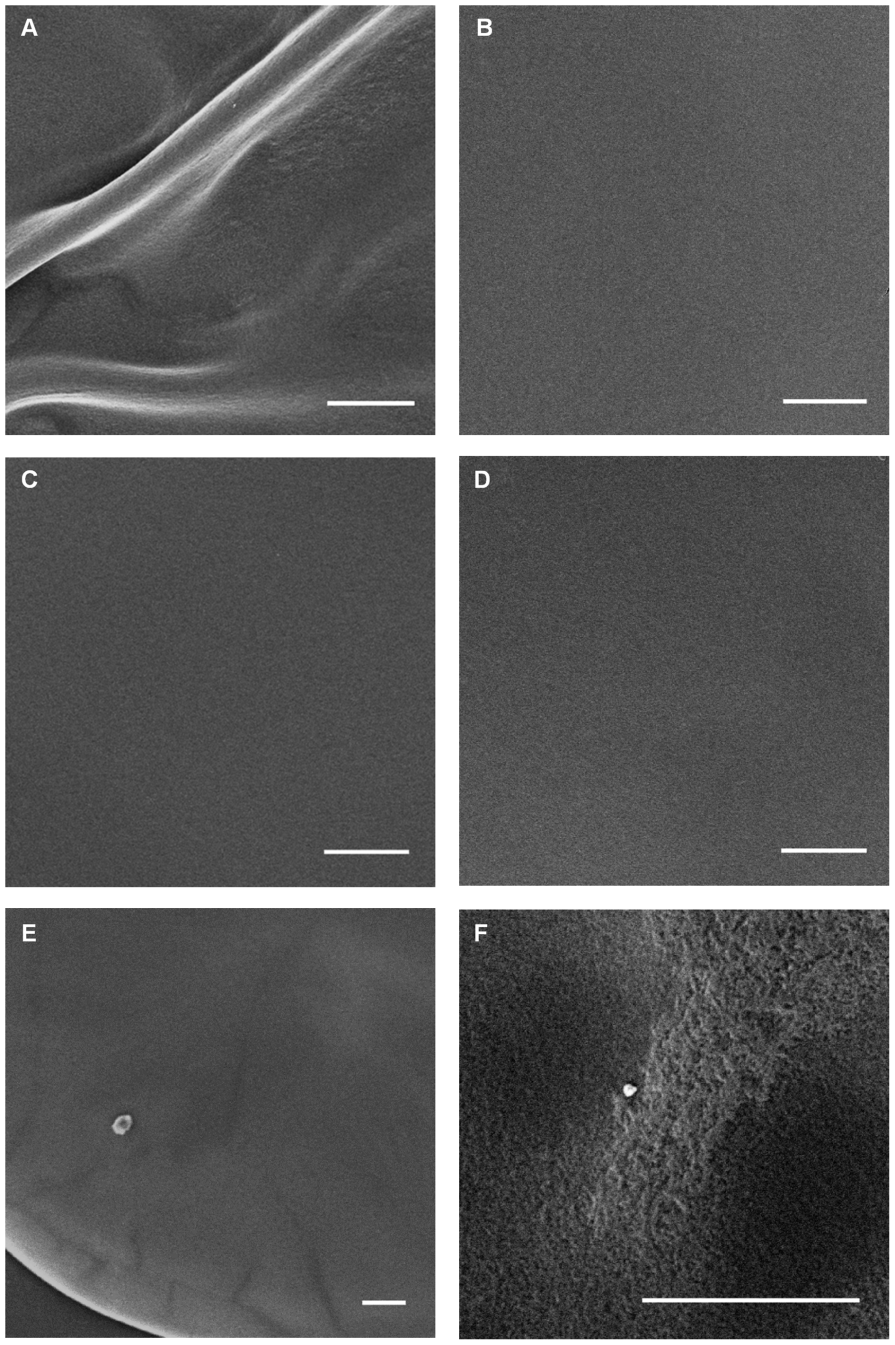
Evidence of three-dimensional structuring of KG3-03 colonies. Whole colonies taken from HI agar plates were vapor fixed with osmium tetroxide and viewed by SEM as described previously [7]. Panels: (A), KG3(R); (B) YJ016; (C), KG4(T); (D) KG3-17; (E, F) KG3-03. All scale bars equal 100 µm. Images in panels E and F are taken from the same colony. All images presented are representative of many images taken for several colonies of each strain. To achieve uniformity, brightness was increased somewhat for images in panels A, B, D and F, while it was decreased for the image in panel C. Contrast was not adjusted for any of the images.

To obtain further evidence consistent with EPS production by KG3-03, colonies of this strain, as well as several others, were subjected to SEM analysis. Previous SEM studies indicated that opaque and translucent variants of *V. vulnificus* have smooth colony surfaces with flat architecture, while EPS-producing rugose variants have wrinkled surfaces and dramatic three-dimensional architectures consisting of pillars and channels [7]. Here, we found that while the smooth flat colony surfaces of the *brpI* mutant KG3-17 (Figure 4, panel D) appeared indistinguishable from those of KG4(T) and YJ016 (panels B and C, respectively), the surface of KG3-03 colonies showed evidence of striations even at relatively low magnification (panel E); upon higher magnification, the striated areas corresponded to partial three-dimensional structuring, which is consistent with production of EPS (panel F). While the EPS isolation results and SEM data support the notion that the *brpJ* gene product was not absolutely required for *brp*-encoded EPS production to occur in strain KG3-03, it was nevertheless found to be necessary for production of a pellicle and robust biofilm formation under the conditions tested (Figure 2A–B).

**Figure 5:**
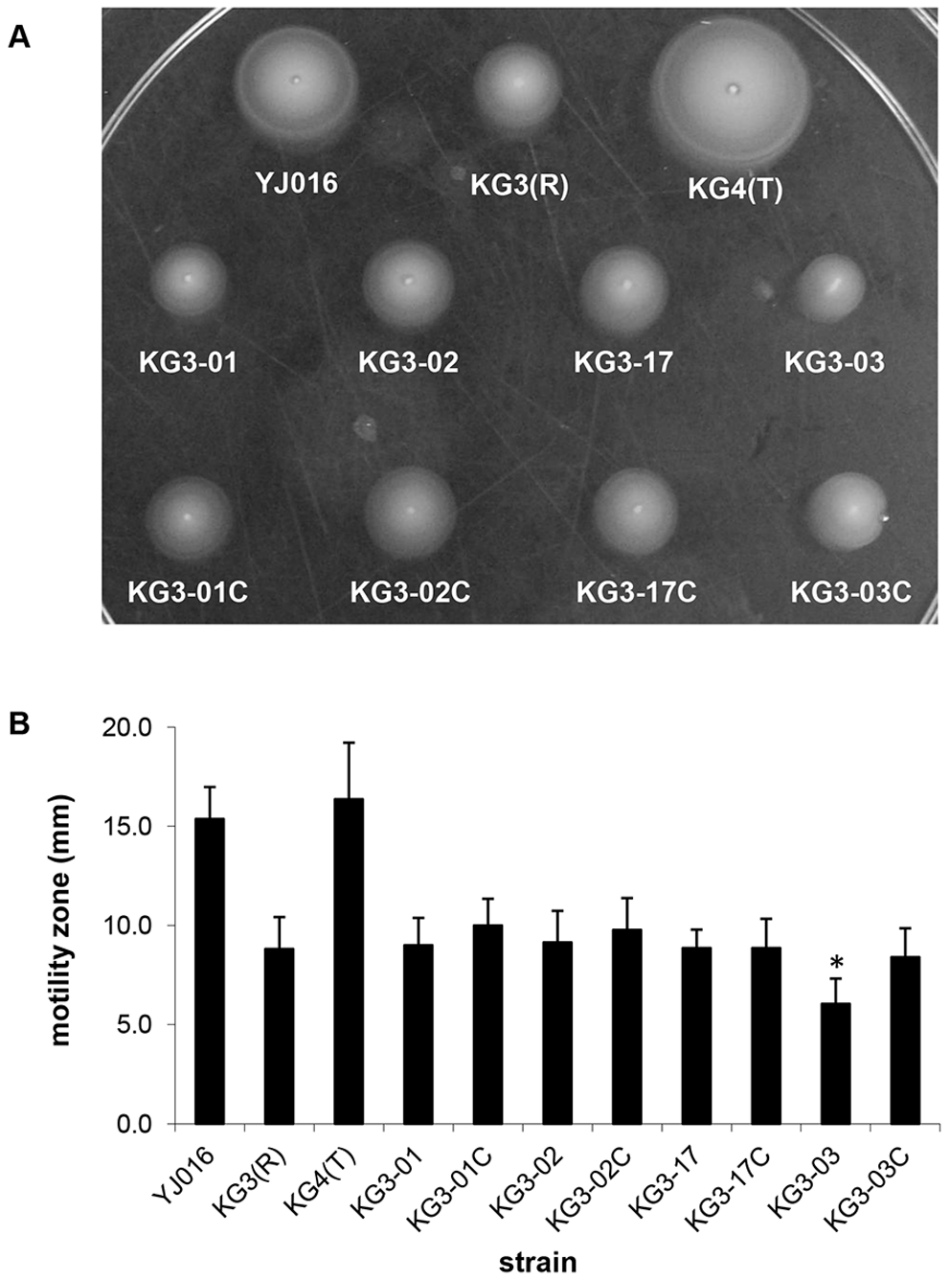
Motilities of individual *brp* mutants are similar to the KG3(R) parent. Motility of opaque, rugose, and translucent control variants, 4 *brp* mutant strains, and the complemented strains on motility agar plates was observed (**A**) and quantified (**B**) for at least 10 replicates of each strain. Averages ± SD are pictured here. Asterisk denotes *p*<0.002 versus KG3(R).

As rugose isolates of *V. vulnificus* are less motile than their opaque or translucent counterparts [6], we measured the motility of each *brp* mutant described here and found that their motilities remained at low levels similar to the rugose parental strain (Figure 5A–B). The motility of KG3-03 was significantly less than KG3(R) (and the other mutants), but we attribute this to its slower growth rate (data not shown). The observation that KG3 mutants retained the reduced motility of KG3(R) suggests that elimination or reduction of EPS production does not appear to affect (i.e., increase) motility in this species.

### Evidence of the *brp* locus being widespread among strains of *V. vulnificus*

Since the *brp* locus is required for rugosity and efficient biofilm formation, it may provide a survival advantage in the environment, and, as such, it would be predicted to be well-conserved among strains of this species. This possibility was tested by screening for the presence of the *brp* cluster in a number of clinical and environmental strains. Linkage PCR was used as previously described [7] to determine that 41 of the 42 *V. vulnificus* strains in our collection contained the *brp* locus (Table 5). The lone exception was the environmental isolate MLT198, which was locked in the translucent phase based on switching assay results (data not shown). To further characterize the *brp* genes in the 41 *brp+* strains, Southern blot hybridization was performed, and it showed that 20 of the strains were of a single 10.5 kb *Pst*I restriction fragment length polymorphism (RFLP) profile while 6 others were of a single 2.7-kb *Pst*I profile (Table 5). The exact profile of the other 15 could not be determined from the available data. All representatives of each RFLP profile group tested showed the ability to switch to the rugose colony type and produce pellicles in culture (data not shown).

**Table 5:** Distribution of the *brp* cluster among *V. vulnificus* strains.

Strain	Linkage PCR^1^	Southern^2^
1001	+	10.5
1003(O)	+	10.5
1004	+	10.5
1005	+	10.5
1007	+	–
1009	+	–
1014	+	–
1456(O)	+	10.5
1456(T)	+	–
1657(O)	+	–
1657(T)	+	–
95-10-15	+	10.5
96-7-155	+	–
ABZ1(R)	+	–
ABZ1(T)	+	–
ATCC 27562	+	10.5
AZ(T)	+	–
BG(R)	+	–
C7184	+	–
CMCP6	+	–
CP Clam 2	+	10.5
CP Mussel 10	+	–
CP Sed 5	+	10.5
F8 Oyster 11(O)	+	10.5
F8 Oyster 11(T)	+	10.5
KG3(R)	+	10.5
KG4(T)	+	10.5
MLT 124	+	10.5
MLT 136(O)	+	10.5
MLT 136(T)	+	10.5
MLT 141	+	10.5
MLT 198	–	–
YJ016	+	10.5
132a1	+	10.5
132z2	+	2.7
212b6	+	2.7
212f15	+	2.7
212s7	+	2.7
342e9	+	10.5
342s6	+	2.7
NOLA18	+	–
212f18	+	2.7

1 + or – indicates the presence or absence, respectively, of all genes in the *brp* cluster.

2 Indicates the size of the band hybridized to a radiolabeled probe specific for *brpC* or *brpI*. A – indicates that either no bands were present or that band size could not be determined.

## Discussion

Here we assessed individual genes within the *brp* cluster for their contribution to rugose colony morphology, biofilm formation, EPS production, and motility. The apparent lack of *brp*-encoded EPS production for the *brpA*, *brpD*, and *brpI* mutants suggests these genes designate required functions. Consistent with this interpretation, the *brpA* and *brpI* genes are predicted to encode glycosyltransferases, which are enzymes that catalyze the sequential transfer of specific individual sugars to an undecaprenyl phosphate carrier lipid during the early steps of polysaccharide synthesis [24]. The *brpD* gene product also appears to be involved in polysaccharide biosynthesis and may play a role in EPS production similar to Wzc, a tyrosine autokinase which is required for assembly of high-molecular-weight CPS and EPS polymers on the bacterial cell surface [7], [24]. The finding here that *brpD* is required for EPS production is consistent with the results for capsule but not K_LPS_ synthesis in *E. coli* K30 *wzc* mutants [24].

On the other hand, the *brpJ* gene appears to be involved in polysaccharide transport as its product has homology to Wzx flippases [7], which are transmembrane proteins that have been proposed to translocate undecaprenyl-phosphate-linked sugar precursors across the cytoplasmic membrane during synthesis of various polysaccharides including certain forms of CPS, EPS and O-antigens of LPS [24], [25]. The reduction, but not elimination, of EPS production in the KG3(R)-derived *brpJ* mutant is reminiscent of the results seen previously for knockout of the *wzxE* flippase gene, which is involved in synthesis of phosphoglyceride-linked and cyclic forms of enterobacterial common antigen in *E. coli* [26], and the production of EPS here suggests a couple of possible explanations. As complementation among Wzx proteins has been demonstrated [27], albeit often at much reduced efficiency [28], it is possible that loss of *brpJ* function can be partially compensated for (either naturally or via suppressor mutation) by a separate Wzx protein encoded by *V. vulnificus*. In this case, a potential candidate may be the *wzx* gene found in the Group I CPS operon [7]. Alternatively, it is known that transport of polysaccharides across the inner membrane of Gram-negatives actually involves two different pathways: one requiring Wzx, which may use an antiport mechanism to flip substrates [29], and the other requiring an ABC transporter [24], [30]. Such movement of individual polysaccharides has long been considered to occur exclusively by one or the other of these pathways; however, using an artificially constructed genetic system, there is now evidence that translocation of at least some lipid-linked oligosaccharides across the inner membrane can be accomplished using either Wzx or ABC transporter function [31]. Therefore another possibility is that an undetermined ABC transporter may also be capable of translocating *brp*-encoded EPS polymers across the inner membrane in *V. vulnificus*.

Based on the results of the EPS isolation for mutants KG3-01, KG3-02 and KG3-17 (Figure 3), we found no evidence of *rbd*-encoded EPS production under the experimental conditions used here. Our results were not surprising given the previous data showing that the *rbd* locus is poorly transcribed under standard lab conditions and is under the control of a two-component sensor kinase/response regulator whose environmental induction signal(s) remains unknown [16]. In any event, the finding that *rbd* polysaccharide does not contribute to rugosity [16] supports the conclusion that the EPS found here on the surface of the *brpJ* mutant KG3-03 is *brp*-encoded.

We also found no evidence that CPS expression is controlled by the *brp* locus, which is in agreement with the previous results of Guo and Rowe-Magnus [12]. Additionally, our results point to another potential mechanism for the creation of phase-locked translucent variants. The transposon mutant TDB3(T), which is derived from the clinical isolate 1003(O), could not be complemented with the *brpIJK* genes and its *wzb* gene remained intact, indicating its translucent phenotype is due to an uncharacterized genetic or epigenetic change. Previous transposon mutagenesis of 1003(O) revealed additional genes that appear to be involved in CPS production in that strain [13], [32]. A mutation in any of these genes or in an additional unidentified gene that affects CPS expression could explain the translucent phenotype of TDB3(T).

Biofilm development occurs in an ordered series of events which begins with initial attachment of planktonic cells to a surface, colonization of that surface, and then development of a three-dimensional architecture [33]. Biofilm formation provides a distinct survival advantage for microbes, because it not only allows them to bind to surfaces that may provide nutrients, but also makes them more resistant to environmental stresses [34], [35]. Consistent with such an advantage we found the *brp* locus to be highly prevalent among *V. vulnificus* strains tested. Further elucidation of the loci that play roles in EPS production in *V. vulnificus* will continue to provide important insights regarding the genetic and physiological bases for biofilm formation by this marine inhabitant and occasional human pathogen.
